# Supplementary material for: The individuality of single-frame functional brain connectivity
Source: bioRxiv. 2026 Jan 5:2026.01.05.675158. Preprint. [Version 1] doi: 10.64898/2026.01.05.675158 (PMC12803208; doi:10.64898/2026.01.05.675158)
Supplement: Supplement 1 [file NIHPP2026.01.05.675158v1-supplement-1.pdf]

bioRxiv preprint doi: https://doi.org/10.64898/2026.01.05.675158; this version posted January 5, 2026. The copyright holder for this preprint (which was not certified by peer review) is the author/funder, who has granted bioRxiv a license to display the preprint in perpetuity. It is made available under aCC-BY 4.0 International license.

Supplementary Material

bioRxiv preprint doi: https://doi.org/10.64898/2026.01.05.675158; this version posted January 5, 2026. The copyright holder for this preprint (which was not certified by peer review) is the author/funder, who has granted bioRxiv a license to display the preprint in perpetuity. It is made available under aCC-BY 4.0 International license.

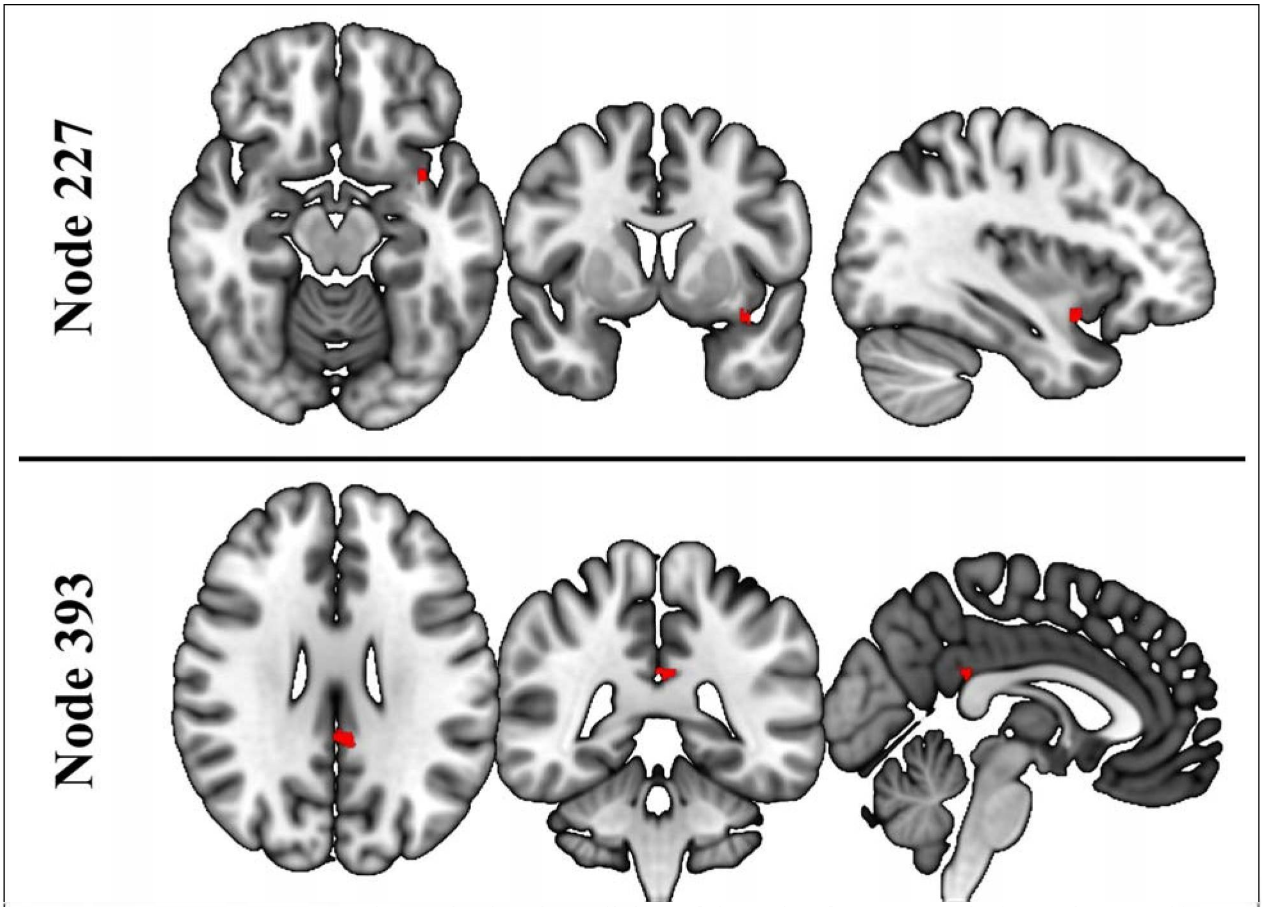

Supplementary Figure 1. Atlas nodes lost in reslicing of the Schaefer 1000-node (17 Subnetwork) atlas. Nodes are highlighted in red. Node 227 was lost in reslicing to 4x4x4mm (for MSC analyses) and 4x4x5 mm (for NCANDA analyses). Node 393 was retained in reslicing to 4x4x4mm but lost in reslicing to 4x4x5mm. Images are in radiological orientation.

751

bioRxiv preprint doi: https://doi.org/10.64898/2026.01.05.675158; this version posted January 5, 2026. The copyright holder for this preprint (which was not certified by peer review) is the author/funder, who has granted bioRxiv a license to display the preprint in perpetuity. It is made available under aCC-BY 4.0 International license.

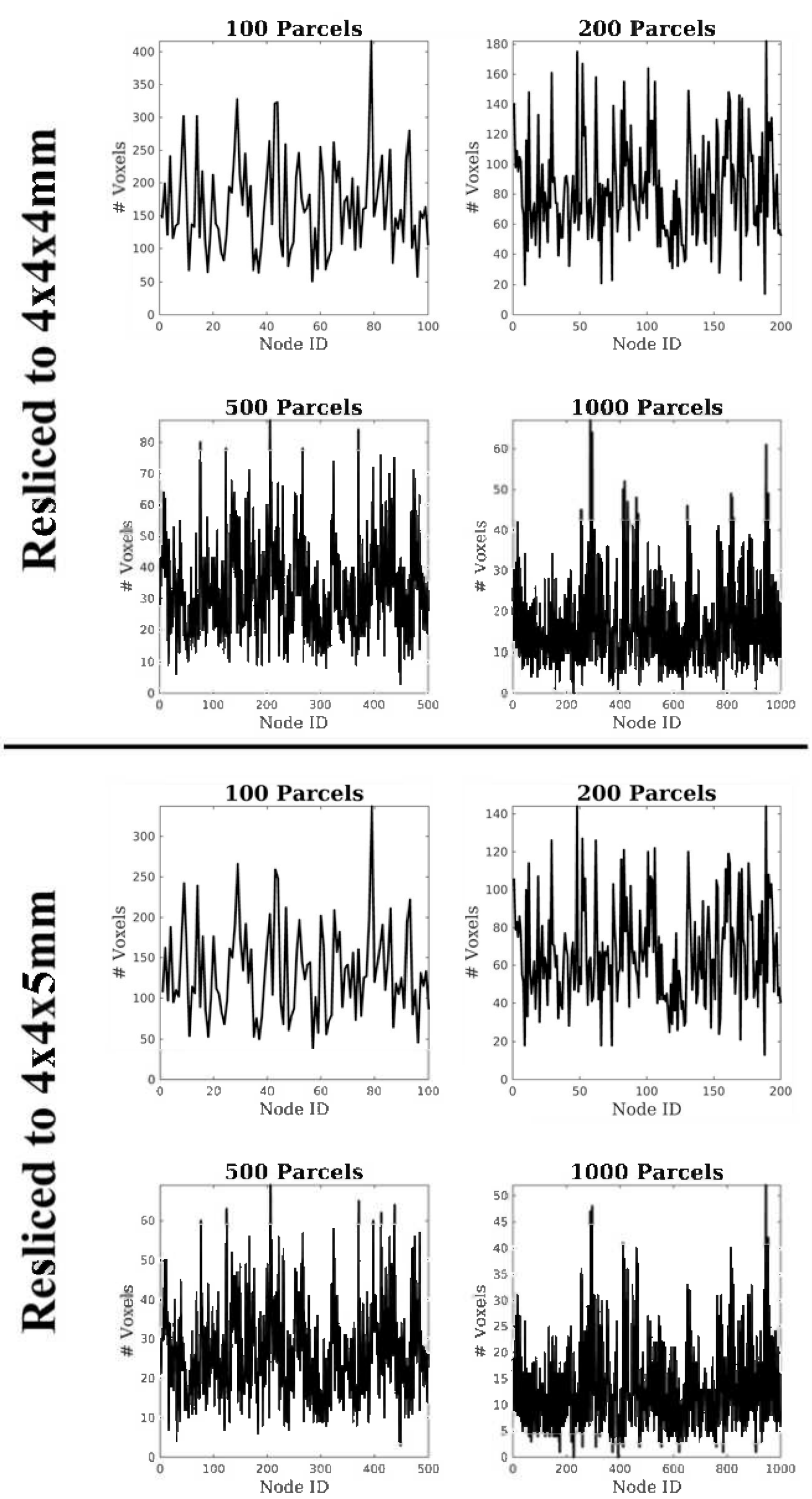

752 **<u>Supplementary Figure 2.</u>** The number of voxels in each node of the Schaefer atlases is shown for both the resliced 4x4x4mm atlas and the 4x4x5mm atlas.

bioRxiv preprint doi: https://doi.org/10.64898/2026.01.05.675158; this version posted January 5, 2026. The copyright holder for this preprint (which was not certified by peer review) is the author/funder, who has granted bioRxiv a license to display the preprint in perpetuity. It is made available under aCC-BY 4.0 International license.

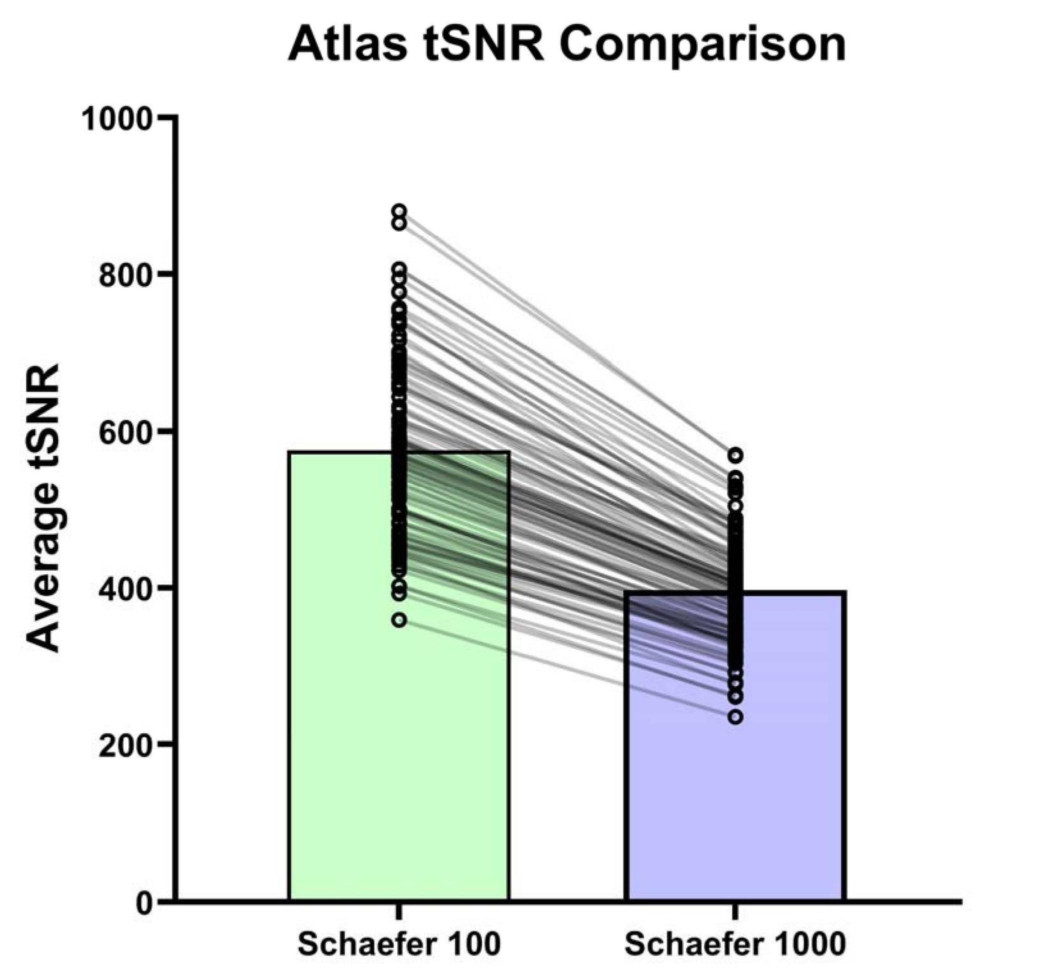

Supplementary Figure 3. Comparison of tSNR (averaged across all nodes) for MSC scans parcellated into the Schaefer 100- versus Schaefer 1000-region atlas. Each dot represents a different person's scan. Lines connecting the two columns indicate the same scan. Lines are semi-transparent to help with following individual lines.

Supplementary Table 1. MSC Individual Volume Identification Accuracy

	1 Database Session	5 Database Sessions	9 Database Sessions
Schaefer 100	41.4 ± 10.2	53.5 ± 8.7	57.4 ± 8.5
Schaefer 200	58.4 ± 12.5	74.1 ± 9.8	78.1 ± 9.2
Schaefer 500	77.7 ± 12.3	90.9 ± 7.2	93.3 ± 6.1
Schaefer 1000	86.8 ± 10.3	96.1 ± 4.9	97.4 ± 4.0

These values correspond to the data shown in Figure 6. Rows indicate different parcellations of the Schaefer atlas (see *Fingerprinting – Atlas Parcellation*) while columns indicate different numbers of database scans used (see *Fingerprinting – Number of Database Scans*). All values indicate percentage of volumes that correctly identified participants for the corresponding atlas and number of database sessions. Error (±) indicates standard deviation of individual scans.
MSC – Midnight Scan Club

bioRxiv preprint doi: https://doi.org/10.64898/2026.01.05.675158; this version posted January 5, 2026. The copyright holder for this preprint (which was not certified by peer review) is the author/funder, who has granted bioRxiv a license to display the preprint in perpetuity. It is made available under aCC-BY 4.0 International license.

Supplementary Table 2. MSC Full Scan Identification Accuracy

	1 Database Session	5 Database Sessions	9 Database Sessions
Schaefer 100	98.7	99.8	100
Schaefer 200	99.9	100	100
Schaefer 500	100	100	100
Schaefer 1000	100	100	100

These values correspond to the data shown in Figure 6. Rows indicate different parcellations of the Schaefer atlas (see *Fingerprinting – Atlas Parcellation*) while columns indicate different numbers of database scans used (see *Fingerprinting – Number of Database Scans*). All values indicate percentage of full scans that correctly identified participants across all iterations of different target scans and subjects.

MSC – Midnight Scan Club

Supplementary Table 3. NCANDA Individual Volume Identification Accuracy

	1 Database Session	3 Database Sessions	5 Database Sessions
Schaefer 100	7.7 ± 5.6	10.4 ± 6.5	11.8 ± 7.1
Schaefer 200	14.2 ± 9.3	20.6 ± 11.0	24.0 ± 11.8
Schaefer 500	27.0 ± 15.0	39.9 ± 16.3	46.3 ± 16.8
Schaefer 1000	37.2 ± 18.1	54.1 ± 18.0	61.6 ± 17.5

These values correspond to the data shown in Figure 6. Rows indicate different parcellations of the Schaefer atlas (see *Fingerprinting – Atlas Parcellation*) while columns indicate different numbers of database scans used (see *Fingerprinting – Number of Database Scans*). All values indicate percentage of volumes that correctly identified participants for the corresponding atlas and number of database sessions. Error (±) indicates standard deviation of individual scans.

NCANDA – National Consortium on Alcohol and NeuroDevelopment in Adolescence.

Supplementary Table 4. NCANDA Full Scan Identification Accuracy

	1 Database Session	3 Database Sessions	5 Database Sessions
Schaefer 100	59.0	81.8	89.3
Schaefer 200	80.1	95.8	97.7
Schaefer 500	93.2	98.9	99.7
Schaefer 1000	95.7	99.6	99.8

These values correspond to the data shown in Figure 6. Rows indicate different parcellations of the Schaefer atlas (see *Fingerprinting – Atlas Parcellation*) while columns indicate different numbers of database scans used (see *Fingerprinting – Number of Database Scans*). All values indicate percentage of full scans that correctly identified participants across all iterations of different target scans and subjects.

bioRxiv preprint doi: https://doi.org/10.64898/2026.01.05.675158; this version posted January 5, 2026. The copyright holder for this preprint (which was not certified by peer review) is the author/funder, who has granted bioRxiv a license to display the preprint in perpetuity. It is made available under aCC-BY 4.0 International license.

NCANDA – National Consortium on Alcohol and NeuroDevelopment in Adolescence

Supplementary Table 5. BNET Individual Volume Identification Accuracy

	1 Database Session
Schaefer 100	6.8 ± 6.4
Schaefer 200	12.2 ± 10.5
Schaefer 500	22.0 ± 16.8
Schaefer 1000	30.6 ± 21.2

These values correspond to the data shown in Figure 6. Rows indicate different parcellations of the Schaefer atlas (see *Fingerprinting – Atlas Parcellation*) while columns indicate different numbers of database scans used (see *Fingerprinting – Number of Database Scans*). All values indicate percentage of volumes that correctly identified participants for the corresponding atlas and number of database sessions. Error (±) indicates standard deviation of individual scans.
BNET – Brain Networks and Mobility

Supplementary Table 6. BNET Full Scan Identification Accuracy

	1 Database Session
Schaefer 100	42.8
Schaefer 200	63.0
Schaefer 500	77.7
Schaefer 1000	83.5

These values correspond to the data shown in Figure 6. Rows indicate different parcellations of the Schaefer atlas (see *Fingerprinting – Atlas Parcellation*) while columns indicate different numbers of database scans used (see *Fingerprinting – Number of Database Scans*). All values indicate percentage of full scans that correctly identified participants across all iterations of different target scans and subjects.
BNET – Brain Networks and Mobility

Supplementary Table 7. MSC Individual Volume Identification Accuracy – No Gray Matter Regression

	1 Database Session	5 Database Sessions	9 Database Sessions
Schaefer 100	42.1 ± 7.7	54.3 ± 9.0	58.1 ± 9.4
Schaefer 1000	86.8 ± 8.3	96.3 ± 4.9	97.5 ± 4.0

Rows indicate different parcellations of the Schaefer atlas (see *Fingerprinting – Atlas Parcellation*) while columns indicate different numbers of database scans used (see *Fingerprinting – Number of Database Scans*). All values indicate percentage of volumes that correctly identified participants for the corresponding atlas and number of database sessions. Error (±) indicates standard deviation of individual scans.
MSC – Midnight Scan Club

bioRxiv preprint doi: https://doi.org/10.64898/2026.01.05.675158; this version posted January 5, 2026. The copyright holder for this preprint (which was not certified by peer review) is the author/funder, who has granted bioRxiv a license to display the preprint in perpetuity. It is made available under aCC-BY 4.0 International license.

Supplementary Table 8. MSC Full Scan Identification Accuracy – No Gray Matter Regression

	1 Database Session	5 Database Sessions	9 Database Sessions
Schaefer 100	97.9	99.4	99.0
Schaefer 1000	99.9	100	100

Rows indicate different parcellations of the Schaefer atlas (see *Fingerprinting – Atlas Parcellation*) while columns indicate different numbers of database scans used (see *Fingerprinting – Number of Database Scans*). All values indicate percentage of full scans that correctly identified participants across all iterations of different target scans and subjects.
MSC – Midnight Scan Club

Supplementary Table 9. MSC Individual Volume Identification Accuracy – High/Low Motion

	High Motion	Low Motion
Schaefer 100	37.7	57.5
Schaefer 200	64.1	78.1
Schaefer 500	85.2	93.4
Schaefer 1000	88.8	97.4

Rows indicate different parcellations of the Schaefer atlas (see *Fingerprinting – Atlas Parcellation*) while columns indicate volumes with high motion versus low motion. Accuracy is based on fingerprinting analyses with 9 database scans. All values indicate percentage of full scans that correctly identified participants across all iterations of different target scans and subjects.
MSC – Midnight Scan Club

Supplementary Table 10. NCANDA Individual Volume Identification Accuracy – High/Low Motion

	High Motion	Low Motion
Schaefer 100	7.3	12.0
Schaefer 200	15.0	24.4
Schaefer 500	30.3	46.9
Schaefer 1000	42.8	62.3

Rows indicate different parcellations of the Schaefer atlas (see *Fingerprinting – Atlas Parcellation*) while columns indicate volumes with high motion versus low motion. Accuracy is based on fingerprinting analyses with 5 database scans. All values indicate percentage of full scans that correctly identified participants across all iterations of different target scans and subjects.
NCANDA – National Consortium on Alcohol and NeuroDevelopment in Adolescence

bioRxiv preprint doi: https://doi.org/10.64898/2026.01.05.675158; this version posted January 5, 2026. The copyright holder for this preprint (which was not certified by peer review) is the author/funder, who has granted bioRxiv a license to display the preprint in perpetuity. It is made available under aCC-BY 4.0 International license.

Supplementary Table 11. BNET Individual Volume Identification Accuracy – High/Low Motion

	High Motion	Low Motion
Schaefer 100	5.2	6.8
Schaefer 200	9.0	12.4
Schaefer 500	17.8	22.2
Schaefer 1000	23.5	31.0

Rows indicate different parcellations of the Schaefer atlas (see *Fingerprinting – Atlas Parcellation*) while columns indicate volumes with high motion versus low motion. Accuracy is based on fingerprinting analyses with 1 database scan. All values indicate percentage of full scans that correctly identified participants across all iterations of different target scans and subjects.

BNET – Brain Networks and Mobility

Supplementary Table 12. MSC Static Network Identification Accuracy

	1 Database Session	5 Database Sessions	9 Database Sessions
Schaefer 100	96.1	99.6	100
Schaefer 200	99.0	100	100
Schaefer 500	100	100	100
Schaefer 1000	99.9	100	100

Percentage of scans for which the individual was correctly identified based on their static network. Rows indicate different parcellations of the Schaefer atlas (see *Fingerprinting – Atlas Parcellation*) while columns indicate different numbers of database scans used (see *Fingerprinting – Number of Database Scans*). All values indicate percentage of full scans that correctly identified participants across all iterations of different target scans and subjects.

MSC – Midnight Scan Club

Supplementary Table 13. NCANDA Static Network Identification Accuracy

	1 Database Session	3 Database Sessions	5 Database Sessions
Schaefer 100	71.7	89.7	93.5
Schaefer 200	83.4	95.3	97.7
Schaefer 500	90.6	97.6	98.5
Schaefer 1000	92.4	98.7	99.3

Percentage of scans for which the individual was correctly identified based on their static network. Rows indicate different parcellations of the Schaefer atlas (see *Fingerprinting – Atlas Parcellation*) while columns indicate different numbers of database scans used (see *Fingerprinting – Number of Database Scans*). All values indicate percentage of full scans that correctly identified participants across all iterations of different target scans and subjects.

NCANDA – National Consortium on Alcohol and NeuroDevelopment in Adolescence

bioRxiv preprint doi: https://doi.org/10.64898/2026.01.05.675158; this version posted January 5, 2026. The copyright holder for this preprint (which was not certified by peer review) is the author/funder, who has granted bioRxiv a license to display the preprint in perpetuity. It is made available under aCC-BY 4.0 International license.

Supplementary Table 14. BNET Static Network Identification Accuracy

	1 Database Session
Schaefer 100	48.3
Schaefer 200	61.0
Schaefer 500	72.5
Schaefer 1000	76.3

Percentage of scans for which the individual was correctly identified based on their static network. Rows indicate different parcellations of the Schaefer atlas (see *Fingerprinting – Atlas Parcellation*). All values indicate percentage of full scans that correctly identified participants across all iterations of different target scans and subjects.

BNET – Brain Networks and Mobility

Supplementary Table 15. Midnight Scan Club Task Identification

	**Faces**	**Words**	**Rest**
**Within Subject**	58.4 ± 10.6	46.4 ± 10.3	60.3 ± 14.4
**Between Subjects**	49.9 ± 9.0	32.1 ± 6.4	51.4 ± 8.5

Values associated with the bar plots in Figure 7. All values indicate percentage of volumes that correctly identified participants for the corresponding atlas and number of database sessions. Error (±) indicates standard deviation of individual scans. By random chance, task identification for individual volumes would be expected to be correct 33.3% of the time.
